# Colonoscopy aspiration lavages for mucosal metataxonomic profiling of spondylarthritis-associated gastrointestinal tract alterations

**DOI:** 10.1038/s41598-023-33597-y

**Published:** 2023-04-28

**Authors:** Ricaurte A. Marquez-Ortiz, Moises Leon, Deisy Abril, Javier Escobar-Perez, Cristian Florez-Sarmiento, Viviana Parra-Izquierdo, Philippe Chalem, Consuelo Romero-Sanchez

**Affiliations:** 1 Bacterial Molecular Genetics Laboratory/LGMB, Vicerrectoría de Investigaciones, Universidad El Bosque, Av. Cra 9 No. 131 A–02, Bogotá, Colombia; 2 Master’s Program in Basic Biomedical Sciences, Faculty of Science, Universidad El Bosque, Bogotá, Colombia; 3 Cellular and Molecular Immunology Group/INMUBO, School of Dentistry, Universidad El Bosque, Av. Cra 9 No. 131 A–02, Bogotá, Colombia; 4 Gastroadvanced, Bogotá, Colombia; 5 Fundación Instituto de Reumatología Fernando Chalem, Bogotá, Colombia; 6 Hospital Militar Central, Rheumatology and Immunology Department, Bogotá, Colombia; 7 Clinical Immunology Group, School of Medicine, Universidad Militar Nueva Granada, Bogotá, Colombia

**Keywords:** Metagenomics, Colonoscopy, Dysbiosis, Spondyloarthritis

## Abstract

The study of the GI-tract microbiota of spondylarthritis (SpA) patients has focused on the analysis of feces samples, that picture mostly the luminal microbiota. The aim of this study was to determine the contribution of mucosal and luminal microbiome to the gut dysbiosis in SpA, using colonoscopy aspiration lavages (CAL), a recent alternative for regional studies of the GI-tract. We analyzed 59 CAL (from sigmoid colon and distal ileum), and 41 feces samples, from 32 SpA patients and 7 healthy individuals, using 16S rRNA gene-targeted metataxonomic profiling. It was found high prevalence of GI-tract manifestations among SpA patients (65.3%). Metataxonomic profiling, confirmed CAL samples from the lower GI tract (colon or ileum) presented a distinctive and undifferentiated bacteriome and separate from that found in feces’ samples or in the beginning of the GI tract (oral cavity (OC)). Lower GI-tract samples and feces of SpA patients exhibited similar behavior to the microbiota of IBD group with reduced microbial richness and diversity, comparing to the healthy controls. Interestingly, it was found increase in proinflammatory taxa in SpA patients, such as *Enterobacteriaceae* family (mostly in the ileum), *Succinivibrio spp.* and *Prevotella stercorea*. Conversely, SpA patients presented significant decrease in the SCFA producers *Coprococcus catus* and *Eubacterium biforme*. Our data support the value of CAL samples for the regional study of GI-tract and contribute with information of potential “disruptor taxa” involved in the GI-tract associated disorders observed in SpA patients.

## Introduction

Microbiome alterations (dysbiosis) in the gastrointestinal (GI) tract have been associated with different pathologies with serious consequences over health and wellness. In some cases, these microbiome alterations can be generated as consequence of systemic and degenerative diseases such as SpA, a group of rheumatic disorders strongly related with the extraintestinal manifestations and gastrointestinal symptoms^1^, being indisputable how the incidence of SpA is increasing in patients with subclinical intestinal inflammation^2^. Even seronegative SpA patients with nonspecific gastrointestinal symptoms, have shown subclinical intestinal inflammations defined by ileocolonoscopic findings^3^.

It has been also reported a causality relation —associated to genetic predisposition—in which a dysbiosis or the mere presence of pathogenic bacteria can trigger an exacerbated immune response promoting the development of several autoimmune disorders such as inflammatory bowel disease (IBD), a manifestation closely related to SpA^4,5^. Numerous studies aimed to describe the gut microbiota composition and its role in the development and progression of SpA^6,7^, however implication of the dysbiosis observed in the gut of these patients is not well understood^8^. Despite controversy, gut microbiota seems to be essential to the development of these pathologies.

The intestinal microbiome has an extremely varied composition with a bacterial diversity ranging from 500 to 1000 species^9^. Implementation of PCR-based massive sequencing technologies (i.e. 16S rRNA gene sequencing) has allowed a wider understanding of microbial communities and contributed to the description of the “healthy” or “unhealthy” microbiome. To study the gut microbial composition, it is possible to use different kinds of samples such as stools or biopsies of the gastrointestinal (GI) tract. The most frequently used is the stool due to easiness and lack of risk during collection^10^, nonetheless, microbiome composition varies according to the location in the GI tract.

Biopsies, laser capture microdissection, luminal brush, among others, are alternatives for the regional study of GI tract that offer the most accurate description of microbiota^10^. However, even though, when an invasive procedure is required (i.e. colonoscopy), a biopsy (or laser capture microdissection) is only requested in the case of abnormal tissue evidence, which makes more difficult the completion of an adequate number of samples in the context of SpA studies. In addition to this, colonoscopies are only prescribed to unhealthy individuals, and considering the risks and complexness of the colonoscopy procedures, to enroll healthy controls always hassles the development of clinical studies. All these reasons have moved gut microbiota studies to the use of feces as a proxy of the intestinal microbiome composition, mostly as a representation of the distal portion of the GI tract^11^. Few studies include more proximal locations like the small intestine due to the difficulty to reach this portion, despite being one of the most compromised tracts in the context of SpA^12^.

In recent years unconventional sampling methods have been proposed, offering similar results to those found in biopsies, such as the use of residues of CAL^13^. Here, we assess the use of CAL from the sigmoid colon and distal ileum —the most proximal part of the small intestine, from ileonoscopies, and compared against feces samples, in order to study the gastrointestinal microbiome in patients with SpA. Our metataxonomic results showed a similar composition of CAL samples from ileum and colon but marked differences with feces. Regardless of the nature of the samples, SpA patients exhibited a significant dysbiosis in different regions of the GI tract, with a mark increased of *Enterobacteriaceae* abundance, that probably originated in the ileum.

## Methods

### Patients, sample collection and handling

Thirty-two patients with SpA, according to ASAS classification and the European Spondyloarthropathy Study Group (ESSG) criteria^14,15^, attended at the Hospital Militar Central and Clinicos IPS in Bogotá, Colombia, were enrolled in a cross-sectional study approved by the institutional ethics committee. Seven healthy volunteer individuals were included as controls for eubiotic microbiota. Additionally, three patients diagnosed with IBD were included to exemplify dysbiosis in the GI tract. Healthy controls aged 18–65 years with lifestyles, socioeconomic status, and professions similar to those of the patients were included. They were questioned regarding the presence of gastrointestinal symptoms (i.e. diarrhea, stools with mucus, hematochezia, daily stool number, abdominal pain and distension) and diet (Supplementary Table S1); clinical examination was also performed. Exclusion criteria were pregnancy, breastfeeding, malignancies, other autoinflammatory or autoimmune diseases, immunodeficiency, chronic pancreatitis, or chronic liver disease and antibiotic treatment in the past 3 months. No control reported diarrhea in the last 4 weeks, 87.6% reported one defection per day, and the remaining 14.3% reported two defecations. Occasional abdominal distension was reported for two controls, and minor food intolerance was reported in 42.9%, mostly due to dairy products (28.6%). Even so, there was no mucus or blood in their stools, and their ileonoscopy did not show evidence of GI tract alterations.

A specific questionnaire was applied asking for gastrointestinal symptoms (Supplementary Table S1), followed by clinical evaluation by a rheumatologist and in patients with ≥ 2 gastrointestinal symptoms, clinical evaluation by a gastroenterologist was also performed. After that, it was defined who had an indication for ileocolonoscopy (with digital chromoendoscopy with magnification at Gastroadvanced Clinic in Bogota, Colombia) and histological analysis. After evaluation by the gastroenterology and before the procedure, ileocolonoscopy benefits and risks were explained to the patients and informed consents were signed. Briefly, a low-volume preparation for colonoscopy was performed with Travad Pik (Sodium Picosulfate 10 mg + light magnesium oxide 3.5 g + citric acid 12 g), to achieve an adequate cleaning of all colonic and distal ileum tracts, measured with the Boston scale (9/9)^16^.

**Figure 1:**
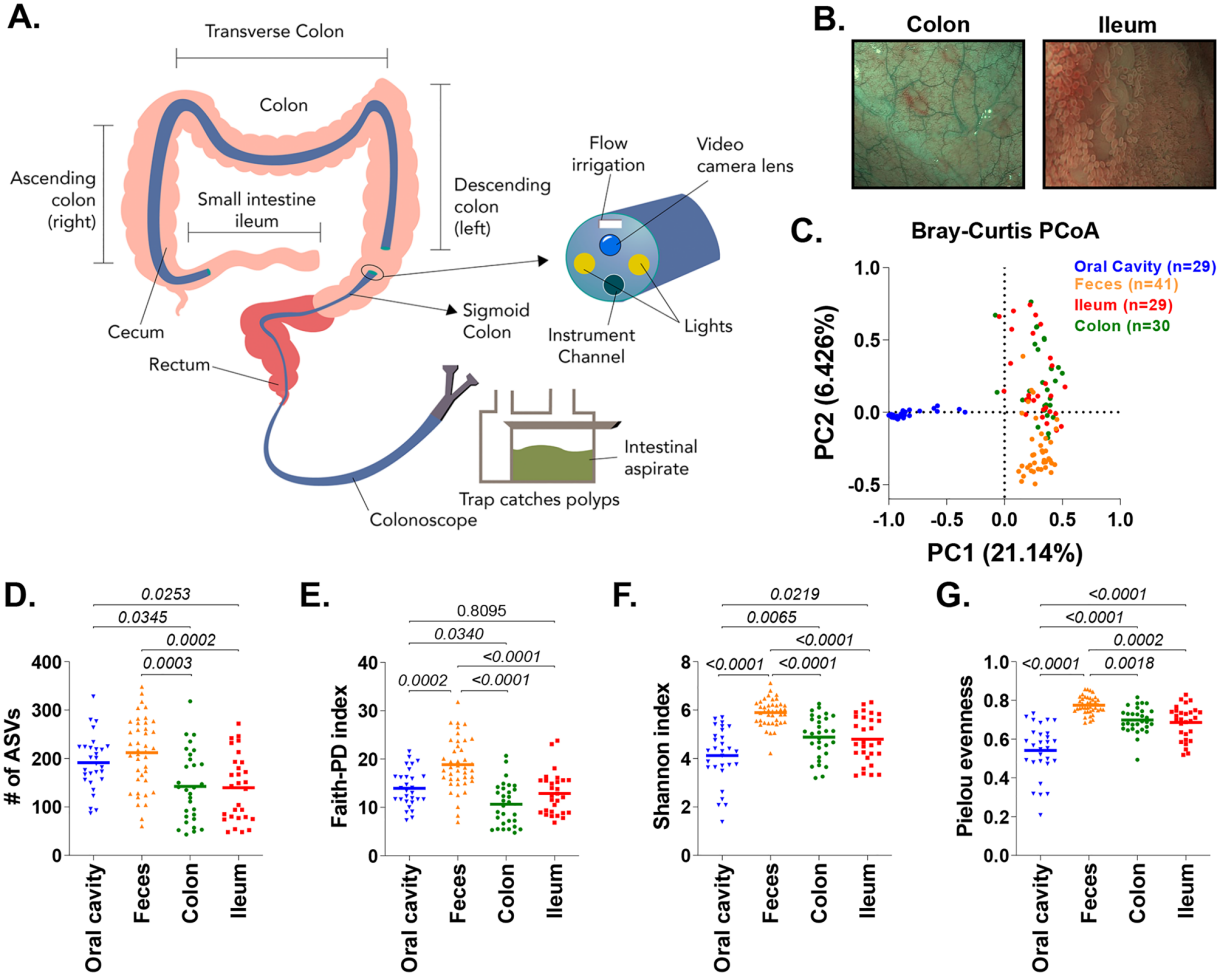
Colonoscopy lavage residues as an alternative for microbiome analysis of the gastrointestinal tract (GI). (**A**) In the colonoscopy, residues from lavages are collected in a polyps’ trap. When the probe reaches the ileum or colon, these zones are washed and the irrigation residues are collected and used for microbiome analysis. Total DNA from ileum and colon lavages, feces and oral cavity (outgroup control) samples was extracted and analyzed by 16S gene-targeted NGS. (**B**) Magnification of colon (left) and ileum (right) areas taken by colonoscopy plus digital chromoendoscopy narrow band imaging from a SpA patient. In the sigmoid colon, superficial ulcers with loss of vascular patter are shown. In the distal ileum, atrophy areas are observed with loss of villi. (**C**) Bray–Curtis dissimilarity Principal Coordinate Analysis (PCoA) based on bacteria community features. (**D**) and (**E**) Richness of bacteria community. *ASV* amplicon sequence variants, *Faith-PD* faith phylogenetic distance. (**F**) and (**G**) Shannon diversity, and Pielou evenness, respectively. Significant p-values (in italics) for Tukey’s multiple comparisons test are shown.

Colonoscopies were performed under sedation with intravenous propofol assisted by an anesthesiologist. All procedures were developed by a gastroenterologist expert in diagnostic and therapeutic endoscopy using EVIS EXERA III CF-HQ190L/I (OLYMPUS) or ELUXEO^®^ 700 Series EC-760ZP-V/L (FUJI). After irrigation with 0.9% NSS (~ 250 mL), aspiration of the left colon and distal ileum was performed with a closed sterile circuit with a ERBE pump (EIP 2 irrigation pump flushes), and the samples were collected with a polyps’ trap (eTrap^®^ BX00711099—US endoscopy) (Fig. 1A–C). Five milliliters of colon (left) and distal ileum aspirates were collected in separate Eppendorf tubes. Patients and healthy individuals also provided a stool sample collected in a sterile container. Both, aspirates (CAL) and stools were kept at − 80 °C before downstream processing.

### DNA extraction and 16S rRNA gene sequencing

Colon and ileum samples (CAL) were centrifuged five minutes at 10,000 rpm and the pellet was used to extract total DNA with the PureLink™ Microbiome DNA Purification Kit (Thermo Fisher Scientific Inc.). Stools’ DNA was extracted using QIAamp PowerFecal Pro DNA Kit (QIAGEN^®^). Extracted DNA was used to prepare 16S rRNA gene amplicon libraries. In brief, a segment spanning the variable regions V3 and V4 in the 16S rRNA gene was amplified by PCR with Herculase II Fusion DNA Polymerase using primers 341F and 805R. These products were used to make Nextera XT Index Kit V2 libraries and sequenced using Illumina MiSeq platform to get 301 bp paired-end reads. After sequencing, the median number of reads was 102,226 (range 56,803–207,845) and reached saturation in rarefaction plots.

### Diversity, abundance and taxonomy analyses

To process the generated libraries the pipeline Qiime 2™^17^ was used as follow. Initially, using DADA2 the last 20 nt in the 3’-end were trimmed away due to low quality, and the reads were cleaned, joined, denoised and clustered as amplicon sequence variants (ASV). It was found 6042 features (ASV) in the libraries with a median frequency of 41,881 (range 9584–68,910). Features were used to determine Shannon, Faith and Richards phylogenetic distance (PD) and Pielou evenness indexes, among others, to evaluate richness, diversity and relative abundance in the study groups. On the other hand, ASVs were classified and assigned to taxonomic levels using Greengenes database (v. 13.5, updated in 2019-05-01)^18^. Taxonomic classification was validated with SILVA 132_99 database (updated in 12-13-2017). For the bioinformatic analysis, due to the physiological differences, we included as an outgroup control oral cavity microbiomes from these patients. Additionally, in spite of the small number of samples, to evidence dysbiosis, microbiomes from IBD patients, collected from CAL and feces were included for the comparisons. However, the statistical analysis was only performed for IBD samples from ileum and colon, where we get at least three samples.

### Statistical analysis

Similarity between groups was evaluated in QIIME2 by means of Unweighted Unifrac distances and principal coordinate analysis (PCoA) with Bray–Curtis index, with PERMANOVA method and computing 1000 permutations. Statistical differences between groups for richness and diversity indexes were assessed using ordinary one-way ANOVA and Tukey’s multiple comparisons test in GraphPad Prism software version 9. In the relative abundance taxonomic analysis, taxa with significant differential abundance between groups were established in STAMP^19^ using White’s non-parametric t-test. Differences between means of average proportions > 0.2% with a p-value < 0.05, were assumed as significant.

### Ethics approval and consent to participate

This study was approved by Corporate Research Ethics Committee of Hospital Militar Central. All participants signed an informed consent to participate and were advised about the risk of the procedures. All experiments were performed in accordance with relevant guidelines and regulations.

## Results

### Sociodemographic characteristics of SpA patients and controls

The group of individuals analyzed in this study was made up of 32 patients with SpA and seven healthy individuals. Of the SpA patients, 56.2% were male, and at the time of the study, 9.4% reported to smoke, 28.1% smoked, and 15.6% considered themselves as passive smokers. Regarding marital and economic status, 56.3% were married (or living with spouse), and the majority were employees (34.4%) or had retiring pension (28.1%). The body mass index (BMI) showed 56.3% were overweight and 12.5% obese, remaining 31.2% presented normal BMI. In the group of healthy controls, 85.7% were women, 14.3% smoke, 42.9% had smoked and 28.6% were passive smokers. According to marital status, 42.8% reported living with spouse, 71.4% were employees, and in this group a normal BMI was observed in 71.4% and the remaining 28.6% were overweight. Regarding eating habits, all controls reported to be omnivorous, whilst 3.1% reported being strict vegetarians in the SpA group. Additionally, in this analysis three patients with IBD (without SpA manifestations) were also included to represent the dysbiotic microbiota. The average age of these patients was 33.2 years (24–54), two men and one woman, all were patients with ulcerative colitis type pancolitis with the Montreal classification for ulcerative colitis of E3S1. Given the mild activity and the extent of the disease, they were under pharmacological treatment, with aminosalicylates at the time the samples were taken; the MAYO clinical activity score was between 3 and 5 denoting mild clinical activity and the MAYO endoscopic score for the 3 patients was 1 with mild endoscopic activity. No patient presented complications derived from ulcerative colitis or required surgery^20,21^.

Focusing on the rheumatological clinical variables in the SpA group, we found that 71.9% of the patients were diagnosed with Ankylosing Spondylitis (AS), 21.9% with Psoriatic Arthritis (PsA) and 6.3% with Reactive Arthritis (ReA). Following ASAS criteria, 9.4% of the patients were found with axial involvement, 25.0% with peripheral involvement, and 65.6% presented axial and peripheral symptoms simultaneously. Infection prior to diagnosis was reported in 9.4% of cases. About musculoskeletal symptoms, the presence of inflammatory lumbar pain was reported in 84.4%, while 15.6% reported mechanical lumbar pain, 87.5% reported enthesitis and arthritis, and 18.8% dactylitis. Biological treatment was reported in 59.4% of SpA patients, with IL-17 inhibitors (9.4%) and anti-TNFα (50%); remaining cases received conventional treatment (40.6%). Reinforcing the role of GI tract manifestations in SpA, 65.3% of these patients reported more than two gastrointestinal symptoms in the last month.

### Colonoscopy aspiration lavages as biological source for the study of mucosal GI tract bacteriome

Using the CAL it was possible to collect 29 and 30 samples from ileum and colon, respectively; also, 41 feces and 29 OC samples were included, the last one like an outgroup control. Metaprofiling of 16S rRNA gene, showed—as expected due to the marked physiological differences—that OC samples clustered as a distant group to the lower GI tract and feces samples in the Bray–Curtis PCoA (Fig. 1C). Though feces and lower GI tract samples grouped nearer, ileum and colon were indistinguishable (multiple linear regression for analysis of variance: p = 0.5691, R square = 0.8609), whilst feces group was slightly shifted to one of the axes and different than ileum and colon (p-values: 0.0051 and 0.0114, respectively). Since stool sweeps along microbes distributed all over the GI tract, this can explain feces differences. Consequently, feces samples revealed a significantly higher species richness, with a mean of 212 ± 73 #ASVs, vs. 142 ± 73 and 140 ± 69, for colon and ileum, respectively (Fig. 1D). Faith phylogenetic distance (richness index based on the phylogenetic tree size) confirmed a significantly richer feces bacterial community, with 18.9 ± 5.4 for feces, vs. 10.6 ± 4.4 and 12.9 ± 4.3 (substitutions/site), for colon and ileum, respectively (Fig. 1E). Despite having bigger ASVs richness (Fig. 1D), OC bacteriome was significantly less diverse, comparing to colon and ileum (Shannon index: 4.9 ± 0.87, 4.8 ± 0.97 and 4.1 ± 1.1, for colon, ileum and OC, respectively) (Fig. 1F). Accordingly, feces samples were the most diverse (Shannon index: 5.9 ± 0.87). In line with that, feces group was the evenest (relative evenness of species), and again lower GI tract samples exhibited intermediate evenness distribution (Fig. 1G). In conclusion, CAL samples from the lower GI tract (colon or ileum) presented a distinctive and undifferentiated bacteriome and separate from than found in feces’ samples or in the beginning of the GI tract such as OC.

### Taxonomic analysis for CAL and feces samples

**Figure 2:**
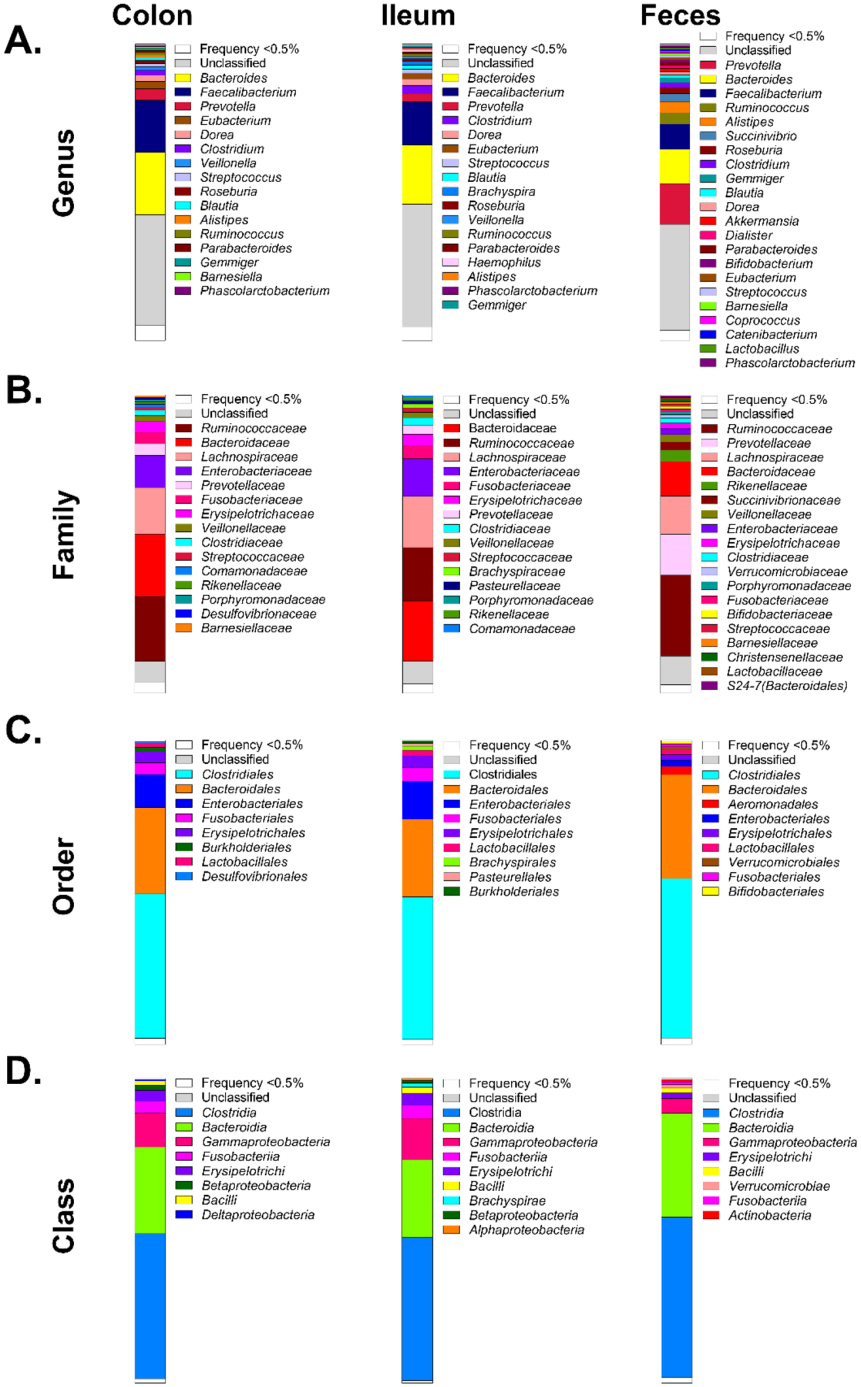
Taxonomic analysis for colon, ileum and feces samples. Colon and ileum exhibit a closer bacteria composition. Bars represent the average relative frequency at different taxonomic levels for each group. Genus (**A**), family (**B**), order (**C**) and class (**D**) levels are shown. Taxa with relative frequency greater than 0.5% are shown. Most abundant taxa are described on the right of each bar, starting on the top with the most abundant features.

Features identified were assessed for taxonomic classification, finding a clear composition signature for the mucosal lower GI tract samples (colon and ileum), composed mostly of *Bacteroides*, *Faecalibacterium*, *Prevotella*, *Eubacterium*, *Dorea* and *Clostridium* genus (Fig. 2A). Although *Bacteroides* and *Faecalibacterium* were also abundant in feces, *Prevotella* showed a marked increase in this group, and in general the predominant bacteriome (conformed by those taxa with a relative frequency > 0.5%) was bigger, including 22 genera, vs. 16 and 17 for colon and ileum, respectively. At the family level, *Ruminococcaceae*, *Bacteroidaceae*, *Lachnospiraceae*, *Enterobacteriaceae*, *Prevotellaceae*, *Fusobacteriaceae*, *Erysipelotrichaceae* were present in the lower GI tract predominant bacteriome. At this taxonomic level, feces samples showed in their predominant bacteriome: *Succinivibrionaceae*, *Verrucomicrobiaceae* and *Bifidobacteriaceae*, among others, that are probably being transported from locations distant from the lower GI tract, or different from the mucosa (luminal circulating bacteria), due to these were not detected as predominant in CAL from colon or ileum (Fig. 2B). Order classification showed *Clostridiales*, *Bacteroidales*, *Enterobacteriales*, *Fusobacteriales*, *Erysipelotrichales* as predominant in the distant GI tract (Fig. 2C). In general, the bigger diversity and richness observed in feces samples can be explained by an increase in families and genera taxa, but not in order and class where the number of taxa identified as predominant in the three groups was similar (Fig. 2C,D).

**Figure 3:**
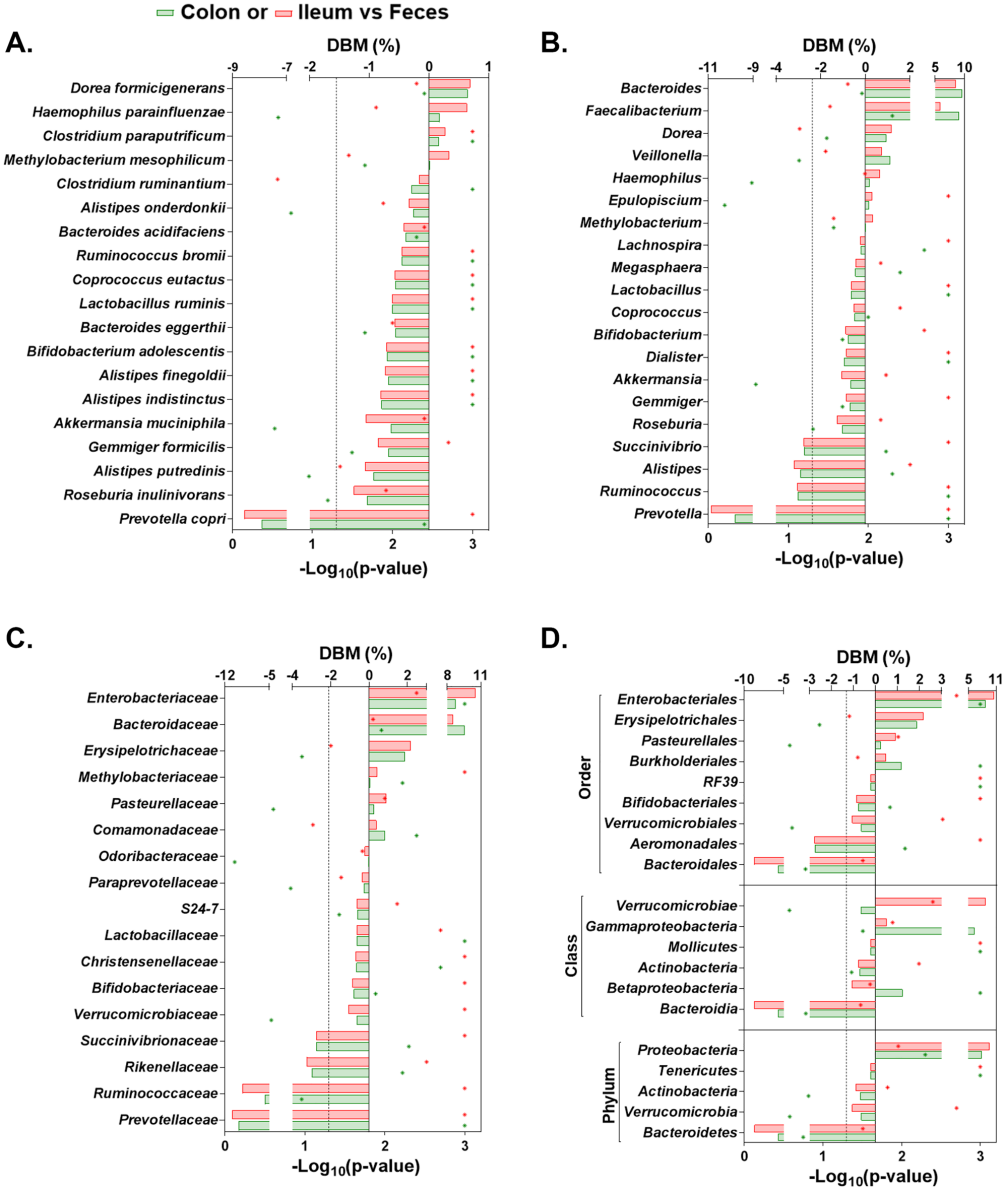
Colon and ileum present a signature bacteria composition with some taxa overrepresented in these regions of the GI tract. Difference between mean relative abundance (DBM, top *x*-axis), for colon (green) or ileum (red) against feces are represented as bars. Positive values indicate those taxa overrepresented in colon or ileum, and negative indicate those overrepresented in feces. Asterisks correspond to p-values (bottom *x*-axis) for White’s non-parametric t-test. Taxa showing DBM > 0.2% and p-value < 0.05 for at least one paired comparison (ileum or colon vs. feces), were included in the plots. (**A**) Species classification. (**B**) Genus. (**C**) Family. (**D**) Order, class and phylum.

Analysis of differential abundance (using differences between means, DBM), did not show statistically significant differences between mucosal colon and ileum bacteriomes, when comparing at species, genus, family, class and phylum levels. However, the order of *Burkholderiales* was more abundant in colon (mean proportion for ileum and colon, respectively: 0.82 ± 1.07% and 1.54 ± 1.52%, p = 0.0499). These results confirm the indistinguishable bacteriome identified in colon or ileum by mean of CAL samples. However, comparing with feces, lower GI tract samples showed enrichment of *Dorea formicigenerans*, *Haemophilus parainfluenzae* (only significant for ileum), *Clostridium paraputrificum* and *Methylobacterium mesophilicum* (Fig. 3A). Colon and ileum samples showed a significant enrichment of *Bacteroides* and *Faecalibacterium* genus; *Enterobacteriaceae* and *Bacteroidaceae* families (Fig. 3C); and *Enterobacteriales* order (Fig. 3D). On the other hand, differential abundance analysis confirmed the enrichment and bigger diversity found in feces (Fig. 3A–D), which is mostly driven by *Bacteroidales* (order), and among these, the *Prevotellaceae* (family) and the *Prevotella* genus, especially *Prevotella copri*. Summarizing, CAL samples from the lower GI tract are useful for the study of mucosal bacteria by mean of 16S rRNA gene targeted strategies, with the depletion of an important number of microorganisms that seems to be unspecifically dragged by feces, from anywhere in the digestive system.

### Use of CAL for the study SpA-associated mucosal GI tract bacteriome

**Figure 4:**
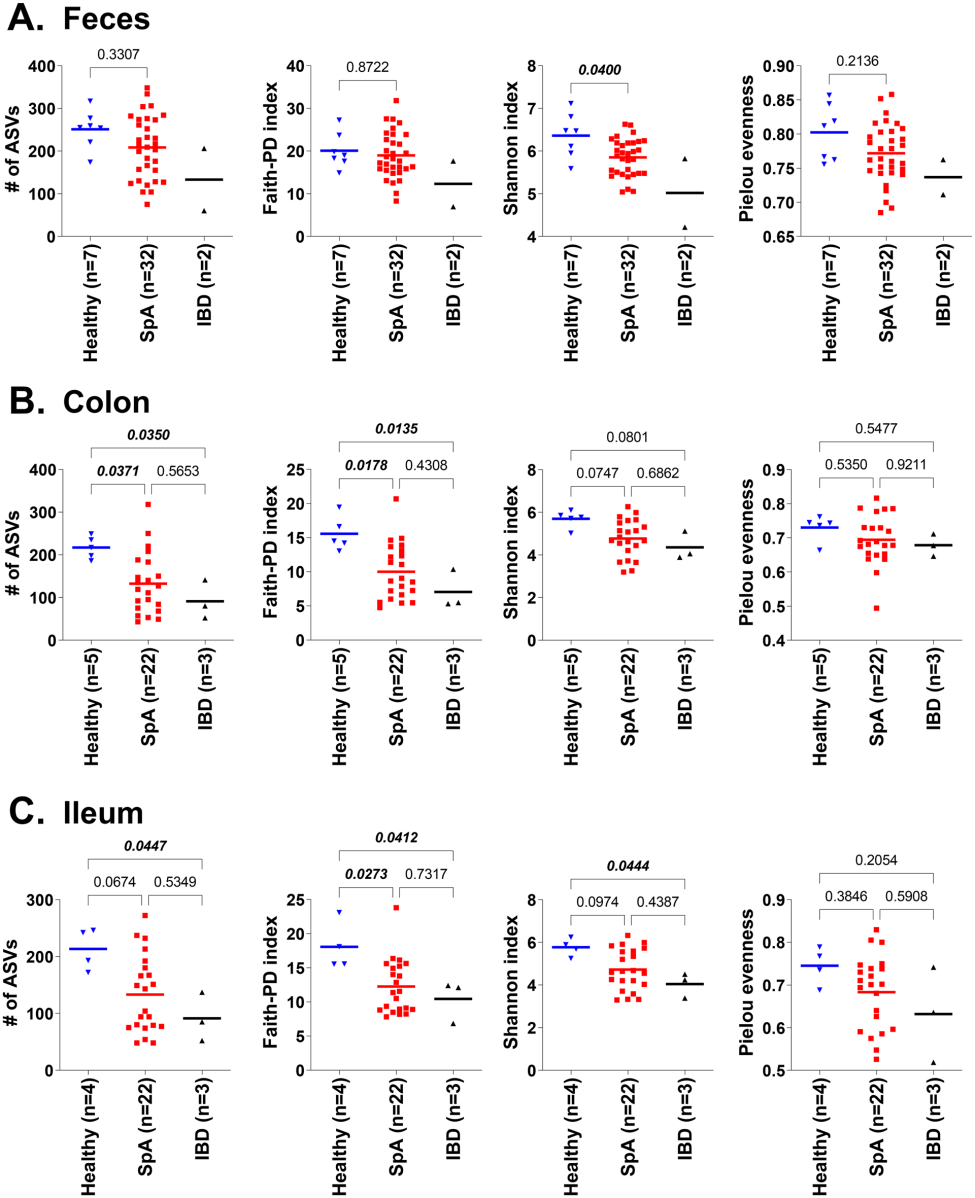
Healthy individuals exhibited the highest bacteria richness and diversity in different areas of the GI tract. Richness (amplicon sequence variants, ASVs), Shannon diversity, Faith phylogenetic diversity (PD) and Pielou evenness, are shown for feces (**A**), colon (**B**) and ileum (**C**) samples. Significant p-values (in bold-italics) for Tukey’s multiple comparisons test are shown. *SpA* spondylarthritis, *IBD* inflammatory bowel disease. For IBD samples, statistical analysis was only performed for ileum and colon, where we get at least three samples. This group was included to exemplify gut dysbiosis.

Libraries of 16S rRNA gene targeted sequencing from feces and CAL samples were used to establish any possible difference between the bacteriome of SpA patients and healthy individuals. Microbiota of feces samples from SpA patients did not exhibit significant differences in the number of ASVs (252 ± 45 and 208 ± 73, SpA vs. healthy, respectively) and Faith-PD (20.1 ± 4.1 and 19.0 ± 5.4, SpA vs. healthy, respectively) richness indexes, compared to those identified in the healthy group (Fig. 4A). Also, Pielou evenness distribution of bacteria population, did not show significant differences between healthy and SpA. Nonetheless, Shannon diversity evaluated in this kind of samples was significantly lower in SpA patients, with a mean of 5.9 ± 0.43 vs. 6.4 ± 0.52 for healthy individuals (p = 0.0400).

Microbiota residing in the colon showed a significant decrease in richness indexes for SpA patients, for both, the mean number of ASVs (217 ± 26 vs. 132 ± 72, healthy and SpA, respectively) and Faith-PD (15.6 ± 2.5 vs. 10.0 ± 4.1) (Fig. 4B). As anticipated for IBD patients, this group showed a significant decrease in richness compared against healthy individuals, but no differences against SpA. Shannon diversity was lower for SpA and IBD patients in this GI tract location, however, differences were not significant, although a possible trend to the decrease cannot be ruled out due to the low p-values (< 0.1). Similar results were found in the ileum; again, richness of bacterium and Shannon diversity were lower for SpA (not significant but with low p-values) and IBD patients, comparing with healthy individuals (Fig. 4C). These results could be explained by insufficiency in the statistics power due to loss of one ileum’s sample in the healthy group.

**Figure 5:**
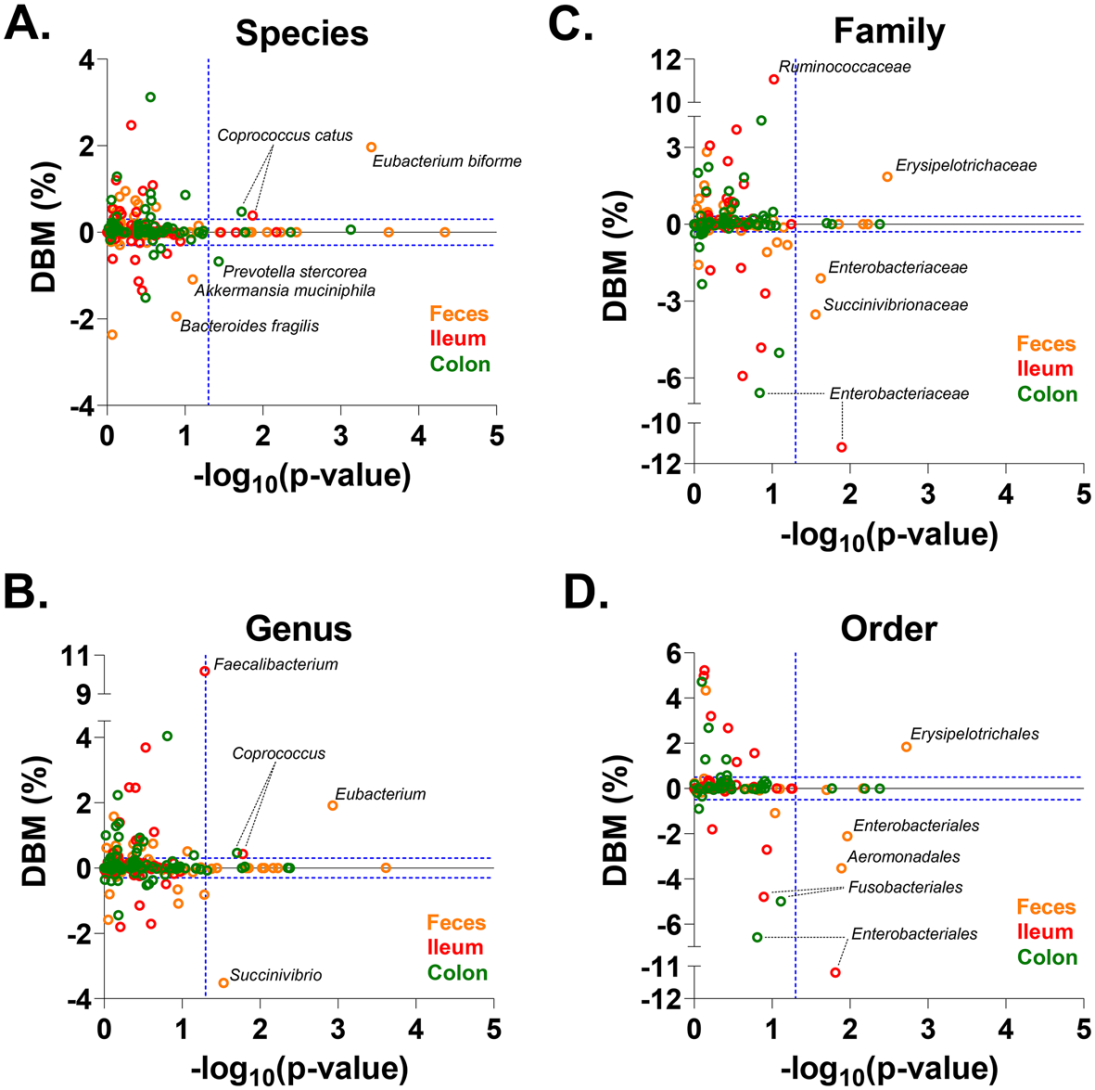
Differences between mean relative abundances (DBM) (*y*-axis) for the control group (healthy individuals) vs. SpA patients, for feces (orange), ileum (red) and colon (green) samples. Positive DBM values indicate those taxa overrepresented in healthy individuals, and negative indicate those overrepresented in SpA patients such as *Prevotella stercorea* in the colon (**A**). In the *x*-axis, p-values for the White’s non-parametric t-test paired comparisons (healthy vs. SpA) are plotted. The DBM arbitrary thresholds (± 0.2%) and the significant p-value threshold (0.05) are shown in dashed blue horizontal and vertical lines, respectively. Taxa with significant differential abundance for species (**A**), genus (**B**), family (**C**) and order (**D**) classifications are shown.

Using the DBM of the relative abundances (percentage in the healthy group minus SpA) we determined taxa showing any disparity between groups (Fig. 5). *Enterobacteriales* order, especially *Enterobacteriaceae* family showed a consistent enrichment in SpA patients (Fig. 5C,D), in both low GI tract locations and feces. Although there was an increase of abundance of around seven percentage points for these taxa in colon samples (bigger than that observed in feces), it was not significant (green dots). It is worth to note that the highest significant increase of these taxa was observed for SpA patients in the ileum (− 11.2%, p = 0.0128). Altogether these suggest that the apparent increase observed in feces is probably driven by an enrichment of *Enterobacteriaceae* family in the lower GI tract of SpA patients, mainly in the ileum.

Healthy individuals showed enrichment of some specific taxa in feces samples, such as *Erysipelotrichales* (order), *Erysipelotrichaceae* (family), *Eubacterium* (genus) and *Eubacterium biforme* (Fig. 5A–D), that were not altered in colon nor ileum, in spite of being part of the abundant microbiome in these locations (see Fig. 2). Similarly, *Aeromonadales* (order), *Succinivibrionaceae* (family) and *Succinivibrio* (genus), were enriched in SpA patients, specifically in feces, but not in colon or ileum, however these taxa were not abundantly found in these other locations. Taking together these results, is suggested that these feces-specific alterations are probably led by bacteria residing in other locations different than sigmoid colon or distal ileum. Moreover, some other taxa such as *Coprococcus* (specifically *C. catus*) and *Prevotella stercorea* (only in colon samples) exhibited reduction and enrichment, respectively, in SpA patients, with slight but significant alterations (Fig. 5A,B), that were only perceptible in colon or ileum CAL samples. These taxa belong to the abundant bacteriome in feces samples, what probably hinders those subtle zone-specific changes observed in colon and/or ileum. In conclusion, our results show that, although SpA-associated dysbiosis can be determined in any type of samples, using CAL it was possible to contribute with evidence of local bacteria taxa alterations in SpA patients.

## Discussion

Use of alternative gut sampling methods is becoming attractive for the deeper analysis of region-specific bacterial compositions. It has been reported that source of nutrients and interaction with the host affect bacteriome composition in the GI tract, increasing abundance and diversity with the progression toward the distal intestine^22^. That is why the use of zone-specific sampling methods is important for the understanding of the dynamics between the host and residing bacteria that promotes pathogenesis. Although biopsies have been recognized like the gold standard for mucosal GI tract regional studies, some limitations such as the low DNA yield (in some cases inadequate for NGS studies), high host DNA contamination, risk of bleeding and infection during collection, and inappropriateness for healthy individuals, increase difficulty of regional GI tract studies. That is why, here we assess the use of CAL for the study of mucosal GI tract alterations in SpA patients. It has been reported CAL generates higher bacterial DNA yields, is applicable to healthy controls and offers minimal differences compared to biopsies^13,23^. Our results contribute to the knowledge of region-specific GI-tract mucosal bacteriome, since up to date (revised on: 03-11-2023) few studies have focused on the use of mucosal rather than fecal samples^13,23–28^, and none of them in the context of SpA associated GI tract alterations.

Our richness, diversity and dissimilarity (PCoA) results demonstrated CAL samples from colon or ileum presented a unique and indistinguishable bacteriome, and different from than found in feces’ samples or in the OC. These results pointed out two important conclusions: first, CAL collection is a good sampling method for the study of mucosal microbiome (that represented by biopsies, laser capture microdissection or CAL), since strong differences were observed when comparing to feces samples that make better representation of the luminal gut bacteriome; Second, CAL samples are not the most appropriate for the identification of changes trough the GI tract progression, due to minimal variations were observed when comparing colon vs. ileum groups (no differences in richness and diversity and only *Burkholderiales* order showed enrichment in colon). Indistinctness of lower GI tract bacteriome (distal ileum vs. sigmoid colon) and difference with fecal bacteriome, have been shown before for biopsies (considered the gold standard)^24,29^. Vaga, et al. found metagenome species richness and diversity did not show significant differences at several mucosal GI tract locations (terminal ileum, transverse colon and rectum) by mean of biopsies; and in a similar way, Zoetendal et al*.* did not find differences in the similarity index using mucosal biopsy samples distributed along the colon^30^. One reasonable explanation for these results using CAL or biopsies, is that changes in mucosal microbiota composition at different locations can be hindered by contamination from the GI luminal fluid through the endoscopic channel^10^. As evidence, the data show that the most predominant bacterial groups in the ileum were anaerobes. It is well-known that most of the ileum's predominant bacterial population are facultative anaerobes, with a small proportion of anaerobes^31^. Nonetheless, to try to reduce contamination, the cecum was washed with NSS (100 mL), and after aspirating the contents, the canal was washed again with NSS (50 mL) prior to cannulating the distal ileum; once inside the distal ileum, an initial aspiration of all the content was performed, which was discarded and irrigation was performed again with NSS (~ 250 ml) and the content was aspirated with a sterile closed circuit, and the samples were collected with a polyp trap.

Nevertheless, our CAL samples successfully pictured the mucosal bacteriome, which is particularly important due to its known direct interaction with epithelial and immune cells^23^. Indeed, SpA patients showed decrease in bacterial richness, and that was more evident at the mucosal bacteriome (ileum and colon). Other studies have pointed to more noticeable differences when using mucosal surfaces instead of fecal samples^27^. Unfortunately, recruitment of healthy individuals consenting collection of colonoscopies (CAL) samples was difficult, reducing our control group, which impacted statistical power for bacterial diversity (Shannon index), even though, SpA patients showed a diversity decrease trend (p < 0.1).

Loss of richness and diversity were explained by enrichment and depletion of some taxa in the SpA patients, highlighting their potential as candidates for future studies. Among them, *Enterobacteriaceae* family showed a consistent enrichment in SpA patients, in both low mucosal GI-tract locations and feces. The highest significant increase for this taxon was observed in the ileum for SpA patients, suggesting that the increase in feces is probably driven the enrichment in the lower mucosal-GI tract, mainly in the ileum, where most of the gut inflammation in axial and peripheral SpA occurs^32^. *Enterobacteriaceae* family has been previously reported as enriched in the fecal microbiota of ankylosing spondylitis, the most representative diagnosis among SpA patients^33^. In addition, some *Enterobacteriaceae* genera such as *Yersinia* spp., *Salmonella* spp. and *Shigella* spp. have been associated to reactive arthritis^34^. It is worth to note that *Enterobacteriaceae* has been shown as a decisive member of the microbial consortia, able to interact with the endogenous microbial community to induce maternal inherited colitis and IBD^35^, and prevention of *Enterobacteriaceae* outgrowth decreased SI damage in murine colitis models^36^. It would be interesting to further investigate *Enterobacteriaceae* to determine members of this family relevant to the interaction with the immune system in our patients, since it has been proved a broad range of bacteria belonging to this family is able to stimulate reactive memory Th1/17 cells, known by its proinflammatory activity (secretion of IFN-γ, IL-17A, and IL-22)^37^, and that could be related to GI-tract or systemic manifestations in SpA patients. Unfortunately, resolution power of the sequencing technology used in this study did not allow to classify SpA-enriched members of this family.

Curiously, we found a strong and significant increase of *Succinivibrionaceae* (and *Succinivibrio spp*), in hand with a non-significant patter of enrichment of *Akkermancia muciniphila* and *Bacteroides fragilis*, in feces samples of SpA patients (Fig. 5). All these taxa have been reported as succinate producers^38^. Remarkably, IBD, colitis and other gut’s microbiota dysbiosis associated pathologies have been linked to succinate accumulation in the gut lumen, which can be either, due to increase in succinate producers and/or decrease in succinate consumers^39,40^. Moreover, Saraiva, et al*.* using mice deficient for the succinate receptor Sucnr1/GPR91, attenuated arthritis development, and reduced traffic and expansion of Th17 cells to the lymph nodes, and conversely, succinate complementation enhanced recruitment and traffic of Th17 and arthritis severity^41^. These findings, and our results of enrichment of succinate-producing bacteria in the lumen of SpA patients, suggest they could be treated with succinate-inflammation targeting therapies, such as Clematichinenoside AR (C-AR), a natural traditional Chinese medicine^42^, or any dietary strategy that reduces circulating succinate. Another taxon involved in succinate production is *Prevotella spp.* which exacerbates mucosal intestinal inflammation, increases succinate and reduces short-chain fatty acids (SCFA) in the GI-tract^43^. Interestingly, we found a significant enrichment of *Prevotella stercorea* in the sigmoid colon of Colombian SpA patients.

Opposite to the increase in succinate-producers, we found a significant decrease of healthy-related taxa, such as *Coprococcus catus* and *Eubacterium biforme* in the mucosal (colon and ileum) and luminal (feces) microbiota of SpA patients, respectively. Both taxa are extensively known by their ability to produce beneficial metabolites and being part of the healthy gut microbiome^44,45^. They produce butyrate and *Coprococcus catus* also produces propionate (SCFAs)^46^. In animal models, dietary supplemented propionate (and some SCFA) has been shown to reduce arthritis severity and joint inflammation^47^, and restores healthy gut microbiota^48^. Consistent with that, butyrate supplementation also impaired arthritis development by promoting production of the metabolite 5-HIAA (5-hydroxyindole-3-acetic acid), by secondary actors in the gut microbiome, and stimulating an immune regulatory state (increasing B_reg_ cells)^49^.

In conclusion, the metataxonomic profiling confirmed CAL samples from the lower GI tract (sigmoid colon or distal ileum), in SpA presented a distinctive bacteriome with similar behavior to the IBD group, with reduced microbial richness and diversity, comparing to the healthy controls. In that sense, refurbishment of healthy microbiota and/or control of metabolites circulation in the GI tract could be a strategy for the treatment of SpA patients, that could complement the conventional or biological treatments to ameliorate symptoms or improve outcomes. This piece of evidence of dysbiosis of SpA patients further supports the importance of exploration of unconventional strategies, such as fiber-rich diets (that increases SCFA), metabolites supplementation or the development of metabolites—or their receptors—targeting therapies. Here, using region-specific microbiota profiling we contribute with information of potential “disruptor taxa” involved in the GI-tract associated disorders observed in SpA patients, that could address the development of these new strategies.

## Conclusions

GI-tract manifestations are highly prevalent among SpA patients. Interaction between gut microbiota and immune system may explain these systemic and local manifestations. Current metataxonomic studies for this disease (and most of GI-tract studies) have focused on the analysis of the luminal microbiome, using feces’ samples, yet cue interactions happen in the mucosa. Here, we implement samples from CAL to represent the mucosal GI-tract and compare against luminal microbiota. To our knowledge, this is the first study of microbial communities in SpA patients using colonoscopy aspiration lavages. Our strategy allowed the inclusion of healthy individuals, not appropriate when using biopsies. We generated important evidence of the SpA luminal and mucosal dysbiosis and identified some “disruptor taxa” previously reported as involved in the activation of inflammatory states, and associated to the enrichment and depletion of some important metabolites, that could address the development or implementation of new strategies for the treatment of SpA. However, to strength these conclusions, future studies should address some limitations in our results, such as the small number of healthy controls and IBD dysbiotic samples.

## Supplementary Information

Supplementary Table S1.

Supplementary Table S2.

## Data Availability

The datasets generated for this study (raw reads) can be found in the NCBI BioProject PRJNA847196 and in the Table S1.

## Abbreviations

SpA: Spondylarthritis
IBD: Inflammatory bowel disease
GI-tract: Gastrointestinal tract
CAL: Colonoscopy aspiration lavages
OC: Oral cavity
Faith-PD: Faith phylogenetic distance
PCoA: Principal coordinate analysis
DBM: Differences between means
SCFA: Short chain fatty acids

## References

[1] Mielants H (1995). The evolution of spondyloarthropathies in relation to gut histology. I. Clinical aspects. J. Rheumatol..

[2] Romero-Sánchez C (2017). Gastrointestinal symptoms and elevated levels of anti- saccharomyces cerevisiae antibodies are associated with higher disease activity in colombian patients with spondyloarthritis. Int. J. Rheumatol..

[3] Mielants H, Veys EM, Cuvelier C, Vos DM (1988). Ileocolonoscopic findings in seronegative spondylarthropathies. Br. J. Rheumatol..

[4] So J, Tam LS (2020). Gut microbiome and its interaction with immune system in spondyloarthritis. Microorganisms.

[5] Foster A, Jacobson K (2013). Changing incidence of inflammatory bowel disease: Environmental influences and lessons learnt from the South Asian population. Front. Pediatr..

[6] Tito RY (2017). Brief report: Dialister as a microbial marker of disease activity in spondyloarthritis. Arthritis Rheumatol..

[7] Breban M (2017). Faecal microbiota study reveals specific dysbiosis in spondyloarthritis. Ann. Rheum. Dis..

[8] Salem F (2019). Gut microbiome in chronic rheumatic and inflammatory bowel diseases: Similarities and differences. United Eur. Gastroenterol. J..

[9] Eisenstein M (2020). The hunt for a healthy microbiome. Nature.

[10] Tang Q (2020). Current sampling methods for gut microbiota: A call for more precise devices. Front. Cell. Infect. Microbiol..

[11] Claesson MJ, Clooney AG, O’Toole PW (2017). A clinician’s guide to microbiome analysis. Nat. Rev. Gastroenterol. Hepatol..

[12] Van Praet L, Jacques P, Van den Bosch F, Elewaut D (2012). The transition of acute to chronic bowel inflammation in spondyloarthritis. Nat. Rev. Rheumatol..

[13] Watt E (2016). Extending colonic mucosal microbiome analysis-assessment of colonic lavage as a proxy for endoscopic colonic biopsies. Microbiome.

[14] Rudwaleit M (2009). The development of Assessment of SpondyloArthritis international Society classification criteria for axial spondyloarthritis (part I): Classification of paper patients by expert opinion including uncertainty appraisal. Ann. Rheum. Dis..

[15] Rudwaleit M (2009). The development of Assessment of SpondyloArthritis international Society classification criteria for axial spondyloarthritis (part II): Validation and final selection. Ann. Rheum. Dis..

[16] Calderwood AH, Jacobson BC (2010). Comprehensive validation of the Boston bowel preparation scale. Gastrointest. Endosc..

[17] Bolyen E (2019). Reproducible, interactive, scalable and extensible microbiome data science using QIIME 2. Nat. Biotechnol..

[18] DeSantis TZ (2006). Greengenes, a chimera-checked 16S rRNA gene database and workbench compatible with ARB. Appl. Environ. Microbiol..

[19] Parks DH, Tyson GW, Hugenholtz P, Beiko RG (2014). STAMP: Statistical analysis of taxonomic and functional profiles. Bioinformatics.

[20] Schroeder KW, Tremaine WJ, Ilstrup DM (1987). Coated oral 5-aminosalicylic acid therapy for mildly to moderately active ulcerative colitis. N. Engl. J. Med..

[21] Silverberg MS (2002). Toward an integrated clinical, molecular and serological classification of inflammatory bowel disease: Report of a working party of the 2005 Montreal World Congress of Gastroenterology. Can. J. Gastroenterol..

[22] Tropini C, Earle KA, Huang KC, Sonnenburg JL (2017). The gut microbiome: Connecting spatial organization to function. Cell Host Microbe.

[23] Miyauchi E (2022). Analysis of colonic mucosa-associated microbiota using endoscopically collected lavage. Sci. Rep..

[24] Vaga S (2020). Compositional and functional differences of the mucosal microbiota along the intestine of healthy individuals. Sci. Rep..

[25] Matsumoto H (2019). Analysis of the colonic mucosa associated microbiota (MAM) using brushing samples during colonic endoscopic procedures. J. Clin. Biochem. Nutr.

[26] Klymiuk I (2021). Characterization of the Luminal and Mucosa-associated microbiome along the gastrointestinal tract: Results from surgically treated preterm infants and a murine model. Nutrients.

[27] Mutlu EA (2014). A compositional look at the human gastrointestinal microbiome and immune activation parameters in HIV infected subjects. PLoS Pathog..

[28] Li G (2015). Diversity of duodenal and rectal microbiota in biopsy tissues and luminal contents in healthy volunteers. J. Microbiol. Biotechnol..

[29] Vasapolli R (2019). Analysis of transcriptionally active bacteria throughout the gastrointestinal tract of healthy individuals. Gastroenterology.

[30] Zoetendal EG (2002). Mucosa-associated bacteria in the human gastrointestinal tract are uniformly distributed along the colon and differ from the community recovered from feces. Appl. Environ. Microbiol..

[31] Villmones HC (2018). Species level description of the human ileal bacterial microbiota. Sci. Rep..

[32] Van Praet L (2013). Microscopic gut inflammation in axial spondyloarthritis: A multiparametric predictive model. Ann. Rheum. Dis..

[33] Klingberg E (2019). A distinct gut microbiota composition in patients with ankylosing spondylitis is associated with increased levels of fecal calprotectin. Arthritis Res. Ther..

[34] Schmitt SK (2017). Reactive arthritis. Infect. Dis. Clin. N. Am..

[35] Garrett WS (2010). Enterobacteriaceae Act in concert with the gut microbiota to induce spontaneous and maternally transmitted colitis. Cell Host Microbe.

[36] Menezes-Garcia Z (2020). Colonization by Enterobacteriaceae is crucial for acute inflammatory responses in murine small intestine via regulation of corticosterone production. Gut Microbes.

[37] Cassotta A (2021). Broadly reactive human CD4+ T cells against Enterobacteriaceae are found in the naïve repertoire and are clonally expanded in the memory repertoire. Eur. J. Immunol..

[38] Fernández-Veledo S, Vendrell J (2019). Gut microbiota-derived succinate: Friend or foe in human metabolic diseases?. Rev. Endocr. Metab. Disord..

[39] Ariake K (2000). Roles of mucosal bacteria and succinic acid in colitis caused by dextran sulfate sodium in mice. J. Med. Dent. Sci..

[40] Morgan XC (2012). Dysfunction of the intestinal microbiome in inflammatory bowel disease and treatment. Genome Biol..

[41] Saraiva AL (2018). Succinate receptor deficiency attenuates arthritis by reducing dendritic cell traffic and expansion of Th17 cells in the lymph nodes. FASEB J..

[42] Li Y (2016). Succinate/NLRP3 inflammasome induces synovial fibroblast activation: Therapeutical effects of clematichinenoside AR on arthritis. Front. Immunol..

[43] Iljazovic A (2021). Perturbation of the gut microbiome by Prevotella spp. enhances host susceptibility to mucosal inflammation. Mucosal Immunol..

[44] Mukherjee A, Lordan C, Ross RP, Cotter PD (2020). Gut microbes from the phylogenetically diverse genus Eubacterium and their various contributions to gut health. Gut Microbes.

[45] El Hage R, Hernandez-Sanabria E, Calatayud Arroyo M, Props R, Van De Wiele T (2019). Propionate-producing consortium restores antibiotic-induced dysbiosis in a dynamic in vitro model of the human intestinal microbial ecosystem. Front Microbiol.

[46] Louis P, Flint HJ (2017). Formation of propionate and butyrate by the human colonic microbiota. Environ. Microbiol..

[47] Friscic J (2021). Dietary derived propionate regulates pathogenic fibroblast function and ameliorates experimental arthritis and inflammatory tissue priming. Nutrients.

[48] Fan Z (2021). Propionate restores disturbed gut microbiota induced by methotrexate in Rheumatoid Arthritis: From clinic to experiments. J. King Saud. Univ. Sci..

[49] Rosser EC (2020). Microbiota-derived metabolites suppress arthritis by amplifying Aryl-hydrocarbon receptor activation in regulatory B cells. Cell Metab..

