# Supplementary material for: Colonoscopy aspiration lavages for mucosal metataxonomic profiling of spondylarthritis-associated gastrointestinal tract alterations
Source: Sci Rep. 2023 Apr 28;13:7015. doi: 10.1038/s41598-023-33597-y (PMC10147911; doi:10.1038/s41598-023-33597-y)
Supplement: Supplementary file 2 — Supplementary Table S2. [file 41598_2023_33597_MOESM2_ESM.docx]

**Supplementary Information**

Table S2. Sociodemographic and clinical variables evaluated.

| **Variable*** | **Description** |
| --- | --- |
| Sex | Male |
|  | Female |
| Civil Status | Single |
|  | Married |
|  | Free union |
|  | Divorced |
|  | Widower |
| Education level | Elementary school |
|  | High School |
|  | Technical |
|  | Bachelor |
| Feeding | Omnivores |
|  | Vegetarians |
|  | Strict vegetarians |
|  | Lactovegetarians |
|  | Ovo vegetarians |
|  | Lacto-ovo vegetarians |
|  | Frugivores |
| Economic activity | Home |
|  | Independent |
|  | Employee |
|  | Pensioner |
|  | Student |
| Housing type | Own |
|  | Leased |
|  | Common |
|  | Accommodation |
| Smokes | Yes |
|  | No |
| Passive smoker | Yes |
|  | No |
| Smoked | Yes |
|  | No |
| Body mass index | Underweight (< 18,5) |
|  | Normal (18,5 – 24,9) |
|  | Overweight (25-29,9) |
|  | Obese (30-34,9) |
|  | Extremely obese (>35) |
| Gastrointestinal symptoms^±^ | Diarrhea |
|  | Stools with mucous/blood |
|  | Abdominal pain |
|  | Abdominal bloating |
|  | None |
| Discomfort from eating food* | Yes |
|  | No |
| Weight loss* | Yes |
|  | No |
| Inflammatory bowel disease* | Yes |
|  | No |
| HLA-B27* | Positive |
|  | Negative |
| Inflammatory back pain* | Age at onset <40 years |
|  | Insidious onset |
|  | Improvement with exercise |
|  | No improvement with rest |
|  | Pain at night with improvement upon getting up |
|  | None |
| Mechanical lumbar pain* | Yes |
|  | No |
| Arthritis* | Yes |
|  | No |
| Enthesitis (heel)* | Yes |
|  | No |
| Uveitis* | Yes |
|  | No |
| Dactylitis* | Yes |
|  | No |
| Psoriasis* | Yes |
|  | No |
| Elevated CRP* | ≧ 3 mg/l |
|  | < 3 mg/l |
| Family history of SpA* | Yes |
|  | No |
| ASAS classification* | Axial |
|  | Peripheral |
| Ankylosing Spondylitis (AS)* | Yes |
|  | No |
| Psoriatic Arthritis (PsA)* | Yes |
|  | No |
| Reactive Arthritis (ReA)* | Yes |
|  | No |
| SpA treatment* | Conventional |
|  | IL-17 inhibitors |
|  | Anti - TNFα |

* Variables used only for SpA patients.

± Variable used as exclusion criteria for healthy controls when two or more GI symptoms were reported.
